# Surveillance to Track Progress Toward Polio Eradication — Worldwide, 2017–2018

**DOI:** 10.15585/mmwr.mm6813a4

**Published:** 2019-04-05

**Authors:** Jaymin C. Patel, Ousmane M. Diop, Tracie Gardner, Smita Chavan, Jaume Jorba, Steven G. F. Wassilak, Jamal Ahmed, Cynthia J. Snider

**Affiliations:** ^1^Global Immunization Division, CDC; ^2^Polio Eradication Department, World Health Organization, Geneva, Switzerland; ^3^Division of Viral Diseases, CDC.

When the Global Polio Eradication Initiative (GPEI) began in 1988, cases of poliomyelitis were reported from 125 countries. Since then, only Afghanistan, Nigeria, and Pakistan have experienced uninterrupted transmission of wild poliovirus (WPV). The primary means of detecting poliovirus is through surveillance for acute flaccid paralysis (AFP) among children aged <15 years with testing of stool specimens for WPV and vaccine-derived polioviruses (VDPVs) in World Health Organization (WHO)–accredited laboratories of the Global Polio Laboratory Network (GPLN) (*1*,*2*). AFP surveillance is supplemented by environmental surveillance for polioviruses in sewage at selected locations. Analysis of genomic sequences of isolated polioviruses enables assessment of transmission by time and place, potential gaps in surveillance, and emergence of VDPVs (*3*). This report presents 2017–2018 poliovirus surveillance data, focusing on 31 countries* identified as high-priority countries because of a “high risk of poliovirus transmission and limited capacity to adequately address those risks” (*4*). Some of these countries are located within WHO regions with endemic polio, and others are in regions that are polio-free. In 2018, 26 (84%) of the 31 countries met AFP surveillance indicators nationally; however, subnational variation in surveillance performance was substantial. Surveillance systems need continued strengthening through monitoring, supervision, and improvements in specimen collection and transport to provide sufficient evidence for interruption of poliovirus circulation.

## Acute Flaccid Paralysis Surveillance

Two surveillance performance indicators assess the quality of AFP surveillance. The first is the nonpolio AFP (NPAFP) rate (the number of NPAFP cases per 100,000 children aged <15 years per year); an NPAFP rate ≥2 is considered sufficiently sensitive to detect circulating poliovirus. The second indicator is the collection of adequate stool specimens (i.e., two stool specimens collected ≥24 hours apart and within 14 days of paralysis onset) and arrival at a WHO-accredited laboratory by reverse cold chain and in good condition (i.e., without leakage or desiccation) from ≥80% of persons with AFP, which ensures sensitivity and provides the specificity to track poliovirus circulation (*2*).

Among the 47 countries in the WHO African Region (AFR), the NPAFP rate in 2017 was 7.0 per 100,000 children aged <15 years, and 92% of AFP cases had adequate stool specimens; in 2018, the NPAFP rate was 5.4 per 100,000 children aged <15 years, and 89% of the AFP cases had adequate stool specimens. Among the 18 high-priority AFR countries assessed, 15 (83%) met both surveillance indicators nationally in 2018, compared with 13 (72%) in 2017 (Table 1). However, national indicators obscure subnational underperformance (Figure). During 2017–2018, no WPV cases were reported in AFR; however, circulating VDPV type 2 (cVDPV2) cases were reported in four countries. In 2017, the Democratic Republic of the Congo accounted for all 22 reported cVDPV2 cases in AFR; in 2018, 65 cVDPV2 cases were reported in the region, including 20 in the Democratic Republic of the Congo, one in Mozambique, 10 in Niger, and 34 in Nigeria (Table 1).

**TABLE 1:** National and subnational acute flaccid paralysis (AFP) performance surveillance indicators and number of confirmed wild poliovirus (WPV) and circulating vaccine-derived poliovirus (cVDPV) cases, by country — 31 Global Polio Eradication Initiative 2018–2020 high-priority countries, World Health Organization (WHO) African, Eastern Mediterranean, South-East Asia, and Western Pacific regions, 2017–2018*

WHO region/Country/Year	No. of AFP cases (all ages)	Regional/National NPAFP rate^†^	Subnational areas with NPAFP rate ≥2 (%)^§^	Regional or national AFP cases with adequate specimens (%)^¶^	Subnational areas with ≥80% adequate specimens (%)	Population living in areas meeting both indicators (%)**	No. of confirmed WPV cases*	No. of confirmed cVDPV cases*^,††^
**2017**								
**African Region**	**31,538**	**7**	**N/A**	**92**	**N/A**	**N/A**	**—^¶¶^**	**22**
Burkina Faso	309	3.6	92	85	77	58	—^¶¶^	—^¶¶^
Burundi	145	2.8	53	83	65	11	—^¶¶^	—^¶¶^
Cameroon	970	9.0	100	86	90	75	—^¶¶^	—^¶¶^
Central African Republic	167	8.0	100	80	43	0	—^¶¶^	—^¶¶^
Chad	703	10.0	100	79	52	56	—^¶¶^	—^¶¶^
Democratic Republic of the Congo	2,148	5.1	100	79	42	32	—^¶¶^	22
Equatorial Guinea	12	2.5	57	17	14	0	—^¶¶^	—^¶¶^
Ethiopia	1,096	2.6	73	86	100	49	—^¶¶^	—^¶¶^
Guinea	452	8.4	100	88	100	86	—^¶¶^	—^¶¶^
Guinea Bissau	83	10.6	100	82	67	35	—^¶¶^	—^¶¶^
Kenya	479	2.3	66	83	68	36	—^¶¶^	—^¶¶^
Liberia	81	4.0	100	81	60	63	—^¶¶^	—^¶¶^
Mali	259	2.9	100	86	89	91	—^¶¶^	—^¶¶^
Mozambique	385	2.8	82	85	55	39	—^¶¶^	—^¶¶^
Niger	682	6.2	100	70	0	0	—^¶¶^	—^¶¶^
Nigeria	16,468	19.6	100	98	100	100	—^¶¶^	—^¶¶^
Sierra Leone	78	2.5	100	77	75	57	—^¶¶^	—^¶¶^
South Sudan	388	7.3	90	84	60	67	—^¶¶^	—^¶¶^
**Eastern Mediterranean Region**	**19,192**	**8.4**	**N/A**	**88**	**N/A**	**N/A**	**22**	**74**
Afghanistan	3,094	20.0	100	94	100	97	14	—^¶¶^
Djibouti	4	1.3	17	100	17	0	—^¶¶^	—^¶¶^
Iraq	699	4.5	95	87	79	74	—^¶¶^	—^¶¶^
Jordan	116	3.3	100	100	100	100	—^¶¶^	—^¶¶^
Lebanon	75	5.3	100	80	83	90	—^¶¶^	—^¶¶^
Libya	88	4.9	100	97	100	100	—^¶¶^	—^¶¶^
Pakistan	10,330	15.0	100	85	100	99	8	—^¶¶^
Somalia	345	5.0	100	99	100	100	—^¶¶^	—^¶¶^
Sudan	570	3.5	100	96	100	100	—^¶¶^	—^¶¶^
Syria	364	4.3	79	76	50	38	—^¶¶^	74
Yemen	713	6.3	100	82	70	68	—^¶¶^	—
**South-East Asia Region**	**43,390**	**8.1**	**N/A**	**86**	**N/A**	**N/A**	—^¶¶^	**—**
Indonesia	1,740	2.4	71	82	47	22	—^¶¶^	—
**Western Pacific Region**	**6,634**	**2.0**	**N/A**	**90**	**N/A**	**N/A**	—^¶¶^	**—**
Papua New Guinea	28	0.9	10	46	15	0	—^¶¶^	—
**2018**								
**African Region**	**24,849**	**5.4**	**N/A**	**89**	**N/A**	**N/A**	**—^¶¶^**	**65**
Burkina Faso	357	4.0	100	86	77	58	—^¶¶^	—^¶¶^
Burundi	123	2.4	53	89	71	11	—^¶¶^	—^¶¶^
Cameroon	778	7.2	100	83	80	73	—^¶¶^	—^¶¶^
Central African Republic	133	6.5	86	68	14	0	—^¶¶^	—^¶¶^
Chad	650	9.0	96	90	78	56	—^¶¶^	—^¶¶^
Democratic Republic of the Congo	2,742	6.6	96	78	58	29	—^¶¶^	20
Equatorial Guinea	30	6.2	86	93	71	0	—^¶¶^	—^¶¶^
Ethiopia	1,083	2.5	73	83	55	49	—^¶¶^	—^¶¶^
Guinea	232	4.2	100	89	88	81	—^¶¶^	—^¶¶^
Guinea Bissau	96	12.0	100	78	44	35	—^¶¶^	—^¶¶^
Kenya	644	3.1	85	87	74	36	—^¶¶^	—^¶¶^
Liberia	72	3.6	100	85	67	43	—^¶¶^	—^¶¶^
Mali	292	3.2	100	87	78	91	—^¶¶^	—^¶¶^
Mozambique	463	3.4	91	87	73	39	—^¶¶^	1
Niger	973	8.5	100	81	75	0	—^¶¶^	10
Nigeria	9,400	10.9	100	95	100	100	—^¶¶^	34
Sierra Leone	114	3.5	100	83	75	57	—^¶¶^	—^¶¶^
South Sudan	430	8.0	100	83	60	67	—^¶¶^	—^¶¶^
**Eastern Mediterranean Region**	**21,834**	**9.5**	**N/A**	**90**	**N/A**	**N/A**	**33**	**12**
Afghanistan	3,376	21.6	100	94	97	98	21	—^¶¶^
Djibouti	0	0	N/A	N/A	N/A	N/A	—^¶¶^	—^¶¶^
Iraq	1,023	6.5	100	90	95	78	—^¶¶^	—^¶¶^
Jordan	115	3.3	100	100	100	100	—^¶¶^	—^¶¶^
Lebanon	89	6.5	100	97	100	94	—^¶¶^	—^¶¶^
Libya	122	6.8	100	96	100	100	—^¶¶^	—^¶¶^
Pakistan	12,190	17.5	100	87	88	99	12	—^¶¶^
Somalia	354	4.9	100	98	100	100	—^¶¶^	12
Sudan	579	3.4	100	97	100	100	—^¶¶^	—^¶¶^
Syria	362	5.5	93	85	86	44	—^¶¶^	—^¶¶^
Yemen	730	6.4	100	92	100	66	—^¶¶^	—^¶¶^
**South-East Asia Region**	**40,493**	**7.6**	**N/A**	**85**	**N/A**	**N/A**	**—^¶¶^**	**1**
Indonesia	1,636	2.3	62	82	44	22	—^¶¶^	1
**Western Pacific Region**	**6,828**	**2.0**	**N/A**	**88**	**N/A**	**N/A**	**—^¶¶^**	**26**
Papua New Guinea	282	8.1	82	46	18	0	—^¶¶^	26

**Abbreviations:** N/A = not applicable; NPAFP = nonpolio acute flaccid paralysis.

* Data as of February 21, 2019.

^†^ Per 100,000 children aged <15 years per year.

^§^ For all subnational areas regardless of population size.

^¶^ Standard WHO target is adequate stool specimen collection from ≥80% of AFP cases, assessed by timeliness and condition. For this analysis, timeliness was defined as two specimens collected ≥24 hours apart (≥1 calendar day in this data set) and within 14 days of paralysis onset. Good condition was defined as arrival of specimens in a WHO-accredited laboratory with reverse cold chain maintained and without leakage or desiccation.

** Percentage of the country’s population living in subnational areas that met both surveillance indicators (NPAFP rates ≥2 per 100,000 children aged <15 years per year and ≥80% of AFP cases with adequate specimens).

^††^ cVDPV was associated with at least one case of AFP with evidence of community transmission and genetically linked. Guidelines for classification of cVDPV can be found at http://polioeradication.org/wp-content/uploads/2016/09/Reporting-and-Classification-of-VDPVs_Aug2016_EN.pdf.

^¶¶^ Dashes indicate that no confirmed cases were detected.

**FIGURE:**
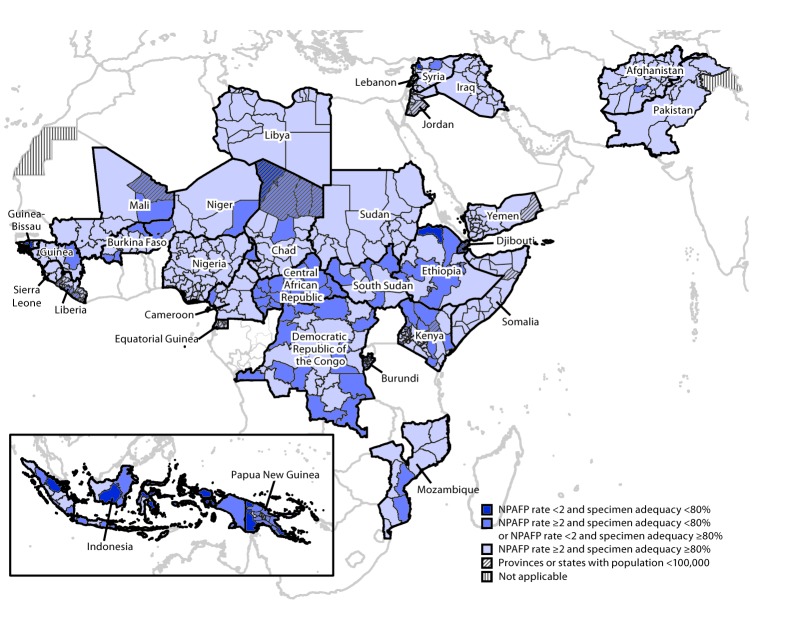
Combined performance indicators for the quality of acute flaccid paralysis surveillance in subnational areas of 31 countries identified as Global Polio Eradication Initiative high-priority countries during 2018–2020 — World Health Organization African, Eastern Mediterranean, South-East Asia, and Western Pacific regions, 2018 **Abbreviation:** NPAFP = nonpolio acute flaccid paralysis.

Among the 21 WHO Eastern Mediterranean Region (EMR) countries, the NPAFP rates in 2017 and 2018 were 8.4 and 9.5 per 100,000 children aged <15 years, respectively, and the respective percentages of AFP cases with adequate stool specimens in 2017 and 2018 were 88% and 90%. In the two countries with endemic WPV transmission, the number of WPV1 cases increased in Afghanistan (from 14 in 2017 to 21 in 2018) and Pakistan (from eight in 2017 to 12 in 2018). In 2017, Syria accounted for all 74 reported cVDPV2 cases in EMR. In 2018, 12 cVDPV cases were reported in Somalia, including five cVDPV2 cases, six cVDPV type 3 (cVDPV3) cases, and one coinfection of both cVDPV type 2 and type 3. Among the 11 high-priority EMR countries evaluated, nine (82%) countries in 2017 and 10 (91%) countries in 2018 met both surveillance indicators nationally; however, as in AFR, national indicators masked subnational underperformance (Table 1) (Figure).

In the WHO Western Pacific Region, 26 cVDPV type 1 (cVDPV1) cases were reported in Papua New Guinea in 2018. Papua New Guinea did not meet either surveillance indicator nationally in 2017, and although the NPAFP rate improved in 2018 (mainly related to implementation of enhanced AFP surveillance as part of the outbreak response), collection of adequate stool specimen remained low. In the WHO South-East Asia Region, one cVDPV1 case was reported in Indonesia in 2018. Although Indonesia met both surveillance indicators nationally in 2017 and 2018, subnational weaknesses in surveillance were substantial (Table 1) (Figure).

## Environmental Surveillance

Environmental surveillance (testing of sewage samples) supplements AFP surveillance by identifying poliovirus transmission in the absence of detected AFP cases (*3*). The number of environmental surveillance sites increased in Afghanistan, Nigeria, and Pakistan from 143 in 2017 to 185 in 2018. Environmental surveillance detected no WPV or cVDPV in Nigeria in 2017; however, 46 cVPDV2 isolates were detected in 2018. Some had been isolated weeks before cases were confirmed. In 2017, four genetic clusters (isolates with ≥95% genetic relatedness) of WPV1 were detected in sewage samples from five provinces in Afghanistan, and seven genetic clusters were detected from 19 districts in Pakistan. In 2018, three WPV1 genetic clusters were detected in sewage samples from seven provinces in Afghanistan and in five clusters from 27 districts in Pakistan. In Pakistan, 16% of sewage samples from 19 districts tested positive for WPV1 in 2017, and 20% from 27 districts tested positive in 2018. Also in 2018, environmental surveillance detected one cVDPV2 isolate in Kenya as well as 19 cVDPV2 and 11 cVDPV3 isolates in Somalia. In Papua New Guinea, environmental surveillance detected seven cVDPV1 isolates from two provinces in 2018. As part of the GPEI’s global environmental surveillance expansion plan,^†^ environmental surveillance is conducted in 44 countries without active WPV transmission, including 24 in AFR.

## Global Polio Laboratory Network

GPLN consists of 146 quality-assured poliovirus laboratories in the six WHO regions. GPLN laboratories implement standardized protocols to 1) isolate and identify polioviruses; 2) conduct intratypic differentiation (ITD) to identify WPV, Sabin (vaccine) poliovirus, and VDPV; and 3) conduct genomic sequencing. Poliovirus transmission pathways are monitored through analysis of the viral capsid protein (VP1) coding region sequences from isolates. Standard timeliness indicators specify that laboratories should report ≥80% of poliovirus culture results within 14 days of specimen receipt, ≥80% of ITD results within 7 days of isolate receipt, and ≥80% of sequencing results within 7 days of ITD result. The combined field and laboratory performance indicator is to report ITD results for ≥80% of isolates within 60 days of paralysis onset in AFP cases. The accuracy and quality of testing at GPLN laboratories are monitored through an annual accreditation program of onsite reviews and proficiency testing (*5*). An accreditation checklist was implemented in 2017 for laboratories testing sewage samples.

GPLN tested 201,546 stool specimens from AFP cases in 2017 and 190,055 in 2018 (Table 2). WPV1 was isolated in specimens from 22 AFP patients in 2017 and 33 patients in 2018. cVDPVs were isolated from 96 patients in 2017 and 104 patients in 2018. GPLN laboratories in all regions met timeliness indicators for poliovirus isolation and ITD. All regions met the overall timeliness indicator for onset to ITD results in both years except the European and Western Pacific Regions in 2018.

**TABLE 2:** Number of poliovirus isolates from stool specimens of persons with acute flaccid paralysis (AFP) and timing of results, by World Health Organization (WHO) region — 2017 and 2018*

WHO region/Year	No. of specimens	No. of poliovirus isolates			% Poliovirus isolation results within 7 days of receipt at laboratory	% ITD results within 7 days of receipt of specimen	% ITD results within 60 days of paralysis onset
		Wild^†^	Sabin^§^	cVDPV^¶^			
**African Region**							
2017	65,245	0	1,663	22	97	80	98
2018	51,292	0	2,547	65	94	98	96
**Americas Region**							
2017	1,755	0	14	0	83	100	100
2018	1,866	0	47	0	86	100	100
**Eastern Mediterranean Region**							
2017	35,602	22	2,521	74	98	99	97
2018	40,419	33	1,749	12	92	99	97
**European Region**							
2017	3,480	0	73	0	83	92	90
2018	3,274	0	71	0	84	92	62
**South-East Asia Region**							
2017	82,292	0	2,251	0	91	96	99
2018	79,566	0	1,970	1	97	100	99
**Western Pacific Region**							
2017	13,370	0	140	0	96	97	90
2018	13,638	0	348	26	97	99	68
Total**							
**2017**	**201,546**	**22**	**6,662**	**96**	**94**	**91**	**98**
**2018**	**190,055**	**33**	**6,732**	**104**	**95**	**99**	**95**

**Abbreviations:** cVDPV = circulating vaccine-derived poliovirus; ITD = intratypic differentiation; VP1 = viral capsid protein.

* Data as of March 4, 2019.

^†^ Number of AFP cases with wild poliovirus isolates.

^§^ Either 1) concordant Sabin-like results in ITD test and vaccine-derived poliovirus screening or 2) ≤1% VP1 nucleotide sequence difference compared with Sabin vaccine virus (≤0.6% for type 2).

^¶^ For poliovirus types 1 and 3, ≥10 VP1 nucleotide differences from the respective poliovirus; for poliovirus type 2, ≥6 VP1 nucleotide differences from Sabin type 2 poliovirus.

** For the last three indicators, total represents weighted percentage of regional performance.

In 2018, South Asia genotype (the only WPV1 genotype circulating globally since 2016) was detected in Afghanistan and Pakistan, with frequent cross-border transmission between the two countries. Compared with the previous report (*1*), sequence analysis indicates a reduction in the number of orphan WPV1 isolates (those with less genetic relatedness [≤98.5% in VP1] to other isolates) from AFP patients, from three in 2017 to zero in 2018, indicating that gaps in AFP surveillance might be closing; sensitive surveillance identifies AFP cases with isolates that are closely related. However, the net genetic diversity of WPV1 isolates in Afghanistan and Pakistan has remained constant for the last 3 years because of the persistent circulation of many poliovirus lineages in the reservoirs of these countries. In 2018, cVDPVs, most with extended divergence from the Sabin strain (genetic relatedness = 94%–98.5% identity), were isolated from stool specimens of AFP patients and from sewage samples, identifying nine cVDPV emergences during 2018 in seven countries (Democratic Republic of the Congo, Indonesia, Mozambique, Niger, Nigeria, Papua New Guinea, and Somalia) (*6*,*7*).

## Discussion

Although most of the 31 GPEI high-priority countries evaluated met national-level AFP performance indicators, considerable variation and deficiencies existed at subnational levels. No substantial improvements were noted in surveillance indicators for these 31 countries from 2017 to 2018. For most of the evaluated AFR countries, the primary deficiency was the low percentage of AFP cases with adequate specimens, which is most often the result of delayed case detection after paralysis onset.

In the three countries with endemic WPV transmission, subnational surveillance performance indicators have been high for several years, even at the district level. In Nigeria, no WPV1 was detected during August 2014–July 2016; however, during August–September 2016, WPV1 cases were detected in Borno State. Effective AFP surveillance did not take place in vast insurgent-held areas of Borno during 2013–2016. Since 2016, more areas have become accessible, and Nigeria has enhanced case detection and reporting by community-based informants residing in currently inaccessible areas (*8*). AFR will be considered for WPV-free certification in early 2020, and careful examination of the extent of quality surveillance will be needed to certify the region WPV-free.

Genomic analyses indicated that the cVDPV1s in Indonesia and Papua New Guinea were circulating several years before detection. Papua New Guinea has experienced chronic national and subnational deficiencies in AFP case detection and adequate specimen collection and transport. Subnational surveillance gaps in Indonesia have been identified previously (*9*). cVDPV outbreaks in regions with endemic polio and those that are polio-free underscore the need to maintain sensitive poliovirus surveillance everywhere to rapidly detect and respond to outbreaks.

AFP surveillance has been complemented by environmental surveillance in high-risk areas, which has allowed detection of cVDPVs before identification of paralyzed patients, as well as documentation of continued circulation of WPV1 in the reservoir areas of Afghanistan and Pakistan despite low-level WPV1 case confirmation. In the long term, continued environmental surveillance will be needed to monitor for poliovirus circulation in high-risk areas.

The findings in this report are subject to at least two limitations. First, issues relating to security, hard-to-reach populations, and other factors could affect AFP surveillance indicators and limit their interpretation. Second, high NPAFP rates do not necessarily indicate highly sensitive surveillance because not all cases reported as AFP cases meet the AFP definition and some actual AFP cases might not be detected by weak surveillance systems.

Strong AFP surveillance, which is essential for global certification of polio eradication, includes timely case detection, notification, and investigation as well as adequate stool collection and transport (*10*). External technical and financial support to enhance surveillance has been provided to all seven countries with cVDPV outbreaks and to the other 24 high-priority countries. The Global Polio Surveillance Action Plan, 2018–2020 (*4*), specifies which tasks are to be undertaken at the country level; support is tailored to countries’ needs. Routine monitoring of AFP surveillance performance indicators at subnational levels and supervision of active surveillance by field personnel are critical to achieving sensitive poliovirus surveillance. Leading up to certification of WPV eradication, integrating AFP surveillance with surveillance for other vaccine-preventable and outbreak-prone diseases will have the advantage of maximizing field surveillance capacity and performance (*10*).

**Summary**

**What is already known about this topic?**

Sensitive acute flaccid paralysis surveillance is the cornerstone of polio eradication programs.

**What is added by this report?**

This report presents 2017–2018 poliovirus surveillance data, focusing on 31 countries identified as high-priority countries by the Global Polio Eradication Initiative. In 2018, 26 (84%) of the 31 countries met acute flaccid paralysis surveillance indicators nationally; however, subnational variation in surveillance performance was substantial, and no improvements were noted from 2017 to 2018.

**What are the implications for public health practice?**

Surveillance systems need continued strengthening through monitoring, supervision, and improvements in specimen collection and transport to provide sufficient evidence for interruption of poliovirus circulation.

## Footnotes

* Afghanistan, Burkina Faso, Burundi, Cameroon, Central African Republic, Chad, Democratic Republic of the Congo, Djibouti, Equatorial Guinea, Ethiopia, Guinea, Guinea Bissau, Indonesia, Iraq, Jordan, Kenya, Lebanon, Liberia, Libya, Mali, Mozambique, Niger, Nigeria, Pakistan, Papua New Guinea, Sierra Leone, Somalia, South Sudan, Sudan, Syria, and Yemen.

† http://polioeradication.org/wp-content/uploads/2016/07/9.6_13IMB.pdf.
